# Lactate Threshold and Psychomotor Fatigue Threshold in Hot Conditions: Suggestions for Soccer Players Participating in the Qatar World Cup 2022

**DOI:** 10.3390/ijerph192417028

**Published:** 2022-12-18

**Authors:** Marek Konefał, Jan Chmura, Małgorzata Charmas, Jadwiga Kotowska, Krzysztof Błażejczyk, Paweł Chmura

**Affiliations:** 1 Department of Human Motor Skills, Wrocław University of Health and Sport Sciences, I.J. Paderewskiego 35, 51-612 Wrocław, Poland; 2 Department of Physiology and Biochemistry, Faulty of Physical Eucation and Health in Biala Podlaska, Józef Piłsudski University of Physical Education in Warszawa, Akademicka 2, 21-500 Biała Podlaska, Poland; 3 Institute of Geography and Spatial Organization, Polish Academy of Sciences, Twarda 51/55, 00-818 Waszawa, Poland; 4 Department of Team Games, Wrocław University of Health and Sport Sciences, I.J. Paderewskiego 35, 51-612 Wrocław, Poland

**Keywords:** heat stress, soccer, choice reaction time (CRT), lactate concentration, incremental test

## Abstract

The study aimed at finding relationships between lactate threshold and psychomotor fatigue threshold during incremental exercise in thermo-neutral climate conditions and conditions for the 2022 FIFA World Cup in Qatar simulated in an environmental test chamber. The study included 24 soccer players aged 21.02 ± 3.22 years old. The following procedures were performed: The incremental exercise test to mark lactate concentration—LA (mmol·l^−1^); Psychomotor test to determine choice reaction time; Designation of the lactate threshold (T_LA_) and psychomotor fatigue threshold (T_PF_). Climate conditions: The procedure was performed twice in the climatic chamber: (1) in thermo-neutral conditions—TNC (ambient temperature 20.5 °C and relative air humidity 58.7%), (2) after 7 days—in Qatar conditions—QC (28.5 ± 1.92 °C) and (58.7 ± 8.64%). It was confirmed that the T_PF_, which reflects the highest efficiency of CNS functioning, occurs at a higher running speed than the T_LA_. The temperature of 28.5 °C with 58.7% humidity, which is the lower limit of heat stress, causes the psychomotor fatigue threshold to appear at a lower running speed than in thermoneutral conditions. The data recorded in this work may help to understand the specificity of physiological and psychomotor reactions to various climatic conditions.

## 1. Introduction

High intensity in modern soccer and the long duration of a match make optimal physical preparation the basis for the effective performance of players [1]. Proper physical preparation can prevent players from becoming fatigued and compromising their performance, especially toward the end of the game. This applies not only to physical performance but also to performance related to the cognitive aspects of the game [2,3]. Therefore, it is important to maintain the optimal efficiency of the players’ analytical and decision-making processes, despite the fatigue growing during the game [4].

The essence of fatigue is the functional changes occurring in players during the match effort, which develop mainly in the motor and nervous systems. Hence, the psychophysiological process that causes fatigue is complex and may result from peripheral and central fatigue. Peripheral fatigue that develops during exercise includes performance-limiting metabolic changes in muscle work [5,6] and is relatively well understood. In contrast, the most common symptoms of central nervous system (CNS) fatigue are inability to maintain the rate of motor neuron activation and a feeling of fatigue but is perhaps less well understood [7].

The two types of fatigue in soccer are related. Research indicates that central fatigue impairs the physical and technical activities of soccer players [8,9]. Few studies [10,11] have revealed that moderate-intensity exercise can improve psychomotor performance, while hard physical work can reduce cognitive performance. At the same time, other authors have indicated that a specific level of peripheral fatigue does not lower or even improve the psychomotor performance of players. This hypothesis is related to the psychomotor fatigue threshold (PFT), i.e., the upper limit of exercise load and fatigue tolerance, accompanied by the shortest choice reaction time (CRT), the highest level of audiovisual differentiation and the most optimal performance. Exceeding PFT leads to the rapid deterioration of these variables [12]. The psychomotor fatigue threshold occurs above the lactate threshold (onset of blood lactate accumulation—OBLA) and is highly correlated with it (r = 0.97) [12]. It seems that the central nervous system has a greater tolerance for fatigue and thus gives greater opportunities to break the fatigue barrier in competitive sports [13,14,15,16].

Most often, studies on central fatigue were carried out before and after exercise [17]. There are few studies that have investigated this fatigue during exercise of increasing intensity [12,13,17], mainly due to methodological problems. Unfortunately, the data available in the scientific literature on this subject are inconclusive [18,19]. In addition, it should be noted that soccer matches and major events, such as the World Cup, take place under different, sometimes very unfavorable, climatic conditions. As we know, environmental conditions are significant modifiers of the physiological and psychomotor reactions of soccer players during a match [20,21,22]. According to [23], playing soccer at an ambient temperature above 28 °C is associated with a high risk of overheating the body, and overheating may lower the level of concentration, psychomotor skills and speed of movement [24,25]. This information becomes important in the context of the 2022 World Cup in Qatar, where the air temperature may exceed 28 °C.

Although the literature includes many studies that take hot conditions into consideration for players [20,21,22,23,24,25], the relationship between performance indicators, such as the lactate threshold (OBLA) and the psychomotor fatigue threshold, has not been studied so far in the context of the hot conditions predicted at one of the most important soccer tournaments in the world. Therefore, the study aimed at finding relationships between lactate threshold and psychomotor fatigue threshold during incremental exercise in thermo-neutral climate conditions and conditions for the 2022 FIFA World Cup in Qatar simulated in an environmental test chamber.

## 2. Materials and Methods

### 2.1. Subjects

The tested players were aged 21.02 ± 3.22 years from a Polish 4th League club. Prior to testing, all athletes were instructed to maintain a normal diet 24 h prior to testing. Body height is 180.22 ± 4.38 cm, body weight 74.20 ± 7.43 kg, VO2max—54.97 ± 5.70 mL·kg^−1^·min^−1^. The load in the training microcycle was five days per week. The study was carried out in November 2018 during the 2018/2019 league season.

The study was conducted in compliance with the Declaration of Helsinki and was approved by the local ethics committee (19/2017). The authors obtained written consent from players or legal guardians to participate in the study. The study was conducted in October 2018 and funded by the National Science Center (grant No. 379,162).

### 2.2. Procedures

#### 2.2.1. Incremental Exercise Test and Tested Parameters

In this research, an exercise test with increasing load was performed on a Cybex 790 T treadmill [26]. According to the classic procedure, the subject started the exercise test at a speed of 8 km·h^−1^, which was increased by another 2 km·h^−1^ every 3 min, until refusal, i.e., reaching the maximum subjective fatigue. During the break after the end of each loading, blood was drawn from a fingertip for the determination of capillary lactate concentration—LA (mmol·l^−1^) (Roche Diagnostics Cobas b 123 analyzer). In addition, during the test, oxygen uptake and VO2 (ml·kg^−1^·min^−1^) were recorded using a mobile ergospirometer CORTEX MetaMax 3b.

#### 2.2.2. Psychomotor Test—Choice Reaction Time

Before the incremental exercise test (Rest) and in the last 30 s of each three-minute load until the maximum load was reached (Max intensity), a psychomotor test was performed. Psychomotor performance was assessed on the basis of choice reaction time (CRT) measurements using an APR measuring instrument (UNI-PAR, Warszawa, Poland). The measurement program was the same for each subject and consisted of 25 visual stimuli: red, green and yellow. Before each CRT measurement, the subject received manual buttons connected to the measuring apparatus. When the monitor emitted red light (10 stimuli), the subject was to press and release the button with the right thumb as soon as possible, when the green light was emitted (10 stimuli)—with the left thumb, while when the yellow color was emitted (5 stimuli), no reaction was required. Visual stimuli were displayed 1.5 m in front of the subject at eye level, according to the procedures described by [12].

#### 2.2.3. Climate Conditions

The tested soccer players and the entire test procedure were performed twice in the Weiss Technik WK-26 climatic chamber (technical specifications: temperature range from −20 to +50 °C, relative humidity range from 10 to 90%, stepless air speed adjustment from 0.2 to 1 m/s): (1) in thermo-neutral conditions—TNC (ambient temperature 20.5 °C and relative air humidity 58.7%), (2) after 7 days—in Qatar conditions (QC) corresponding to the average of the maximum values of ambient temperatures (28.5 ± 1.92 °C) and relative air humidity (58.7 ± 8.64%) recorded in the last 10 years on November 21 in Doha, Qatar (date and place of the World Cup in 2022) [27]. Before starting the tests, the soccer players, after entering the climatic chamber, adapted for 10 min in a sitting position to the conditions in the chamber. The tests were carried out in a certified human performance laboratory.

#### 2.2.4. Designation of the Lactate Threshold and Psychomotor Fatigue Threshold

The lactate threshold (T_LA_) was defined as the exercise load at which blood LA levels reached 4 mmol·l^−1^ using the relationship between the log LA concentration and exercise intensity [28,29]. The psychomotor fatigue threshold (T_PF_) was defined as the running speed corresponding to the shortest choice of reaction time [12].

#### 2.2.5. Statistical Analysis

The normality of the data distribution was checked using the Shapiro–Wilk test. The data are presented as means with standard deviations (SD). To compare the values of the parameters tested obtained before the incremental exercise test (Rest), in T_LA_, in T_PF_ and after the incremental exercise test (Max intensity), a 2-way analysis of variance for repeated measures was used where the first factor was thermos-neutral conditions and the second conditions in Qatar. Differences between pairs of means were checked using Fisher’s least significant difference (LSD) test. A *p* < 0.05 was used as the level of significance [30]. All statistical analyses were conducted using STATISTICA ver. 13.3 software (from StatSoft. Inc., Tulsa, OK, USA).

## 3. Results

The analysis of running speed (V) in the progressive test showed that the psychomotor fatigue threshold of T_PF_ in both climatic conditions occurred above the lactate threshold. Under thermoneutral conditions, T_LA_ occurred at the speed of 13.04 ± 2.09 km·h^−1^, T_PF_—at 15.00 ± 1.44 km·h^−1^ and the maximum intensity was achieved by the soccer players at a speed of 17.58 ± 1.44 km·h^−1^. The increase in running speed between the indicated values was statistically significant (*p* ≤ 0.05). In Qatar conditions T_LA_ occurred at a speed of 13.37 ± 1.90 km·h^−1^, T_PF_—at 14.25 ± 1.36 km·h^−1^ and the maximum intensity was achieved by the players at a speed of 17.08 ± 1.02 km·h^−1^. In addition, the increase in running speed between the indicated values was statistically significant (*p* ≤ 0.05). There were no significant differences between the climatic conditions at any measurement point (T_LA_, T_PF_, max intensity).

The concentration of LA on the lactate threshold (T_LA_) in both conditions, according to the adopted research methodology, was 4 mmol·l^−1^. On the other hand, the level of LA concentration on the psychomotor fatigue threshold (T_PF_) under TNC conditions was 6.20 ± 2.59 mmol·l^−1^, and in QC—5.10 ± 1.91 mmol·l^−1^. This difference was statistically significant (*p* ≤ 0.05).

**Figure 1:**
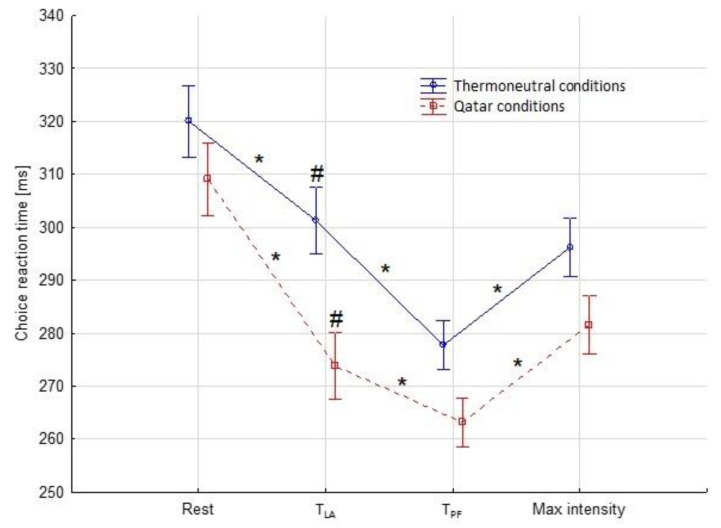
Choice reaction times in subsequent measurement times (means ± SD). Rest—before the incremental exercise test. T_LA_—lactate threshold. T_PF_—psychomotor fatigue threshold. Max intensity—maximum load reached during incremental exercise test. * statistical significance between subsequent measurement times in incremental exercise test. # statistical significance between climate conditions.

The statistical analysis of the choice reaction time revealed no interactions or effects between the studied groups. However, an effect was observed between the climate conditions (thermos-neutral, Qatar conditions)—(F(1) = 6.00, *p* = 0.018) and between subsequent measurement times in the incremental exercise test (Rest, T_LA_, T_PF_, Max intensity)—(F(1) = 46.35, *p* = 0.001). Significant differences (*p* ≤ 0.05) as determined by a post-hoc test between subsequent measurement times in the incremental exercise test and between climate conditions are shown in Figure 1.

## 4. Discussion

The study aimed to find relationships between lactate threshold (T_LA_) and psychomotor fatigue threshold (T_PF_) during incremental exercise in thermo-neutral climate conditions and conditions for the 2022 FIFA World Cup in Qatar simulated in an environmental test chamber.

Research on exercise-related central fatigue in the past has mainly focused on motor system performance and less on the cognitive aspects of fatigue [31]. However, the important role of cognitive functions and central fatigue in endurance exercises, often performed in difficult environmental conditions, has been known for many years and presented in various scientific aspects, e.g., in the form of “spiritual biographies” of athletes [32]. The research goal proposed in this study is part of the innovative, widely discussed in the literature, scientific search for the optimal intensity of exercise at which the speed of cognitive functions improves [33,34]. This aspect and its relationship to peripheral fatigue are less clear.

The changes in the choice reaction time of athletes who performed the psychomotor test during incremental exercise recorded in our research show the relationship between T_LA_ and T_PF_ very clearly. From the beginning of the effort, the choice reaction time has been systematically shortened; even after exceeding the T_LA_, soccer players continue to significantly improve their results until they achieve the best results on the T_PF_. This result is not surprising, as it is in line with the Chmura and Nazar study published in 2010 on a group of young soccer players. These authors claim that the best-choice reaction time (CRT) is achieved by players at the running speed of the psychomotor fatigue threshold. In their research, T_PF_ appears at a running speed of 14.48 ± 0.19 km·h^−1^, which is significantly higher than the speed at 13.39 ± 0.16 km·h^−1^ [12]. For comparison, our research also indicates a significant difference in running speed between T_LA_ and T_PF_ (in which players achieve the best results in choice reaction time); in thermo-neutral conditions (TNC), it is a difference of 1.96 km·h^−1^ and in Qatar conditions (QC)—0.88 km·h^−1^.

At this point, it is worth emphasizing that taking into account the climate conditions factor in our research, we found that this factor differentiates the relationship between T_LA_ and T_PF_. These differences relate to the running speed at which individual thresholds appear. At an ambient temperature of 20.5 °C and a relative air humidity of 58.7% (TNC), T_LA_ occurs at a lower speed—13.04 ± 2.09 km·h^−1^, and T_PF_ at a higher speed—15.00 ± 1.44 km·h^−1^ (difference 11%), compared to the expected ambient temperature at the World Cap in Qatar 28.5 ± 1.92 °C and relative air humidity of 58.7 ± 8.64% (QC), where T_LA_ occurs at a speed of—13.37 ± 1.90 km·h^−1^, and T_PF_ at—14.25 ± 1.36 km·h^−1^ (only 6% difference). This percentage difference is mainly due to the shift of the T_PF_ towards the lower running speed in QC. It seems that the ambient temperature predicted at the World Cup in Qatar (28.5 °C) does not significantly affect peripheral fatigue, but at the same time promotes a significant acceleration of the appearance of T_PF_, which is beneficial from the point of view of the technical and tactical activity of soccer players [20]. Therefore, it can be concluded, complementing the study [23], that the ambient temperature not significantly exceeding 28 °C does not disturb the homeostasis of the organism and is beneficial for psychomotor abilities (which indicates a better CRT in hotter conditions at the T_LA_). Additionally, our observations support the point of view that moderate-intensity exercise has a positive effect on the speed of undertaking cognitive tasks, regardless of the type of task [33]. At the same time, they are complemented by quite ambiguous results concerning the influence of intensive exercise on cognitive functions, indicating the occurrence of the inverted U-letter effect in the speed of undertaking tasks in both analyzed climatic conditions [33].

In the context of the above observations, the results of the LA concentrations recorded in our research are very interesting. It is surprising that in the conditions of a high risk of heat stress (QC), the studied players obtained significantly lower LA values at the psychomotor fatigue threshold compared to the values recorded in the TNC. This may be due to the lower running speed on the psychomotor threshold under QC. It is lower by 0.75 km·h^−1^. Lower exercise intensity generates less fatigue, and therefore, the body responds with lower lactate levels. At the same time, the temperature difference between the climatic conditions included in our research, amounting to only 8 °C, may be too small to significantly affect the physiological reactions of players [27]. Moreover, a temperature of 28.5 °C with humidity below 60% is the lower limit from which the high risk of heat stress begins [23]. It is likely that a higher value of ambient temperature would cause a deeper physiological response [35]. All the reactions observed in these studies should be analyzed in the context of the match effort. This study especially concerns the conditions under which the World Cup in Qatar will be performed. From a practical point of view, players in higher ambient temperatures subconsciously reduce the intensity of exercise, while increasing the effectiveness of technical activities in the game. This cognitive aspect often determines the final success of the team [2,20,22].

The limitation of the research was the performance of the experiment in a climatic chamber, i.e., conditions not specific for soccer players. The effort during the research was made on a treadmill and not on the pitch, which is also not specific to soccer. Other physiological and biochemical parameters (e.g., heart rate, ventilation, adrenaline, noradrenaline, serotonin and dopamine) that would refine the interpretation of the results were not taken into account. Hence, these results should be treated with caution. Future research should cover different age groups and different levels of training experience in a soccer-field setting. In addition, it would be worth analyzing psychomotor parameters under the influence of higher heat stress.

## 5. Conclusions

It was confirmed that the psychomotor fatigue threshold, which reflects the highest efficiency of CNS functioning, occurs at a higher running speed than the lactate threshold.

The temperature of 28.5 °C with 58.7% humidity, which is the lower limit of heat stress, causes the psychomotor fatigue threshold to appear at a lower running speed than in thermoneutral conditions.

Although the conditions simulated in the climate chamber predicted in Qatar are considered to be heat stress, the temperature difference to the thermoneutral conditions of only 8 °C may be too small to significantly affect the physiological and psychomotor responses of players.

The data recorded in this work may help to understand the specificity of physiological and psychomotor reactions to various climatic conditions. This will enable coaches and training staff to adjust their training sessions and match assumptions in more demanding environmental conditions.

## Data Availability

The data presented in this study are available on request from the corresponding author due to restrictions privacy.
